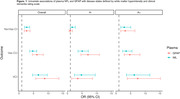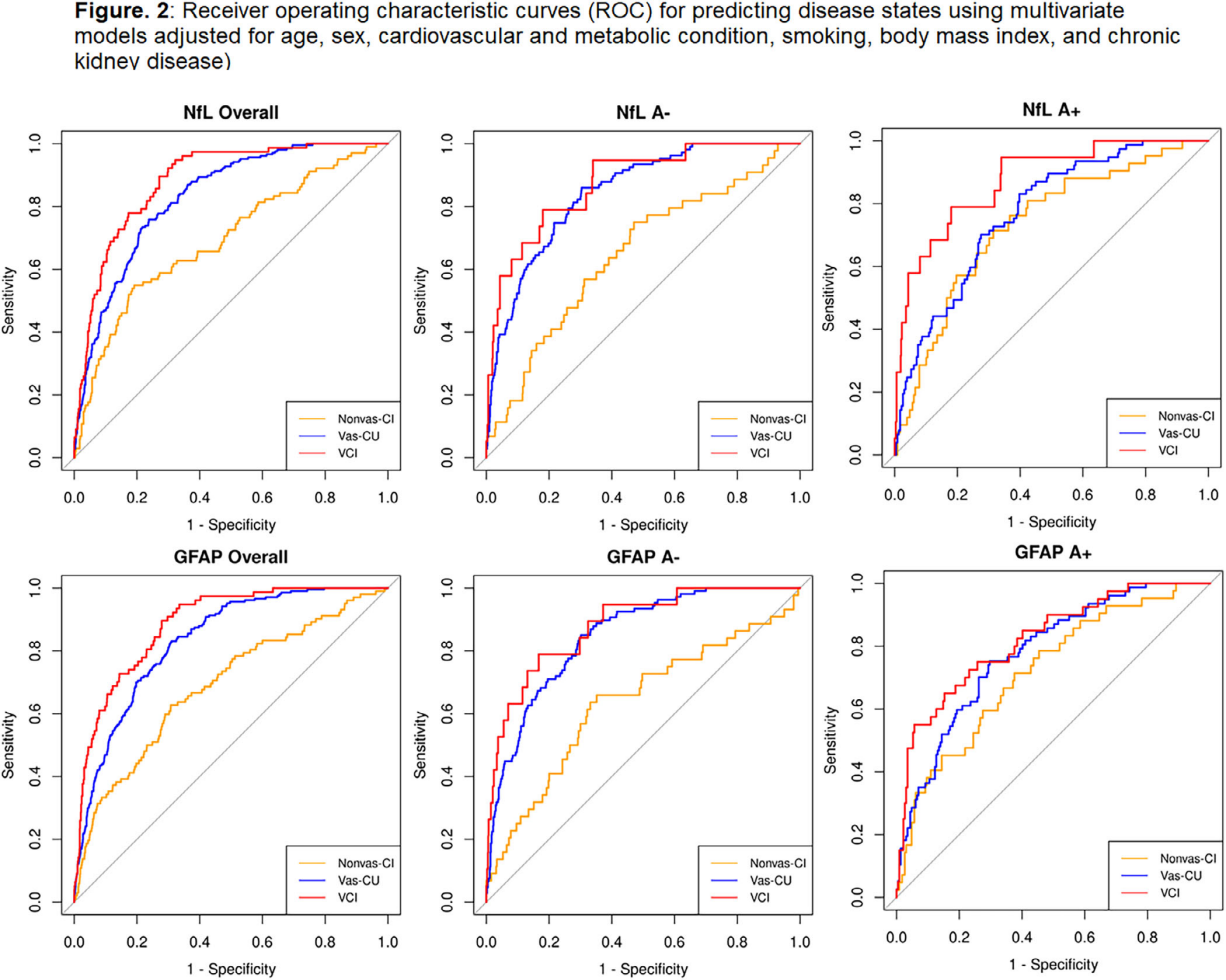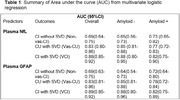# Plasma NfL and GFAP for predicting VCID brain changes in the population

**DOI:** 10.1002/alz.090142

**Published:** 2025-01-09

**Authors:** Sheelakumari Raghavan, Ekaterina I. Hofrenning, Angela J. Fought, Alicia Algeciras‐Schimnich, David S. Knopman, Clifford R. Jack, Ronald C. Petersen, Val J. Lowe, Jonathan Graff‐Radford, Prashanthi Vemuri

**Affiliations:** ^1^ Mayo Clinic, Rochester, MN USA; ^2^ Department of Quantitative Health Sciences, Mayo Clinic, Rochester, MN USA; ^3^ Department of Radiology, Mayo Clinic, Rochester, MN USA; ^4^ Mayo Clinic Alzheimer's Disease Research Center, Rochester, MN USA; ^5^ U.S. Advisory Council on Alzheimer’s Research, Care, and Services, Washington, DC USA; ^6^ Department of Neurology, Mayo Clinic, Rochester, MN USA; ^7^ Mayo Clinic College of Medicine, Rochester, MN USA

## Abstract

**Background:**

Vascular contributions to cognitive impairment and dementia (VCID) are often comorbid with Alzheimer’s disease and increase the risk of dementia. Blood‐based biomarkers may be promising for identifying individuals at high risk for VCID due to small vessel disease (SVD).

**Method:**

We included 1709 participants from the Mayo Clinic Study of Aging who had concurrent MRI, plasma neurofilament light chain (NfL) and glial fibrillary acidic protein (GFAP) measured on the HD‐X Simoa Quanterix platform, and clinical dementia rating scale (CDR). We categorized participants into SVD vs. not using WMH/TIV≥1.3% which corresponds to Fazekas>=2 cutoff and cognitively impaired (CI) vs. not using CDR≥0.5. We obtained four groups using SVD and cognitively normal and impaired classifications: cognitively unimpaired without SVD (CU), CU with SVD (Vas‐CU), CI with SVD (VCI), and CI without SVD (Non‐vas‐CI). Univariate and multivariable logistic regression models were used to obtain odds ratios, confidence intervals and ROC curves to evaluate the association between plasma markers and each disease group compared to CU. We also evaluated these analyses in amyloid positive and negative participants.

**Result:**

There was a high proportion of CU (Vas‐CU/(CU+Vas‐CU)=18%) and CI (VCI/(VCI+ Non‐vas‐CI)=47%) participants with SVD in the population‐based sample. Higher plasma levels were associated with greater vascular burden and differed by amyloid status (Figure 1). ROC analyses showed that both NfL and GFAP provided discrimination between Vas‐CU and CU groups (AUC=0.83) as well as VCI and CU groups (AUC=0.88) (Figure 2, Table 1). Though GFAP and NfL are non‐specific and may also reflect non‐vascular damage, we found that these plasma markers did not clearly separate NonVas‐CI from the CU group in the overall cohort (AUC=0.69). When amyloid status was available, the discriminative power of NfL and GFAP in separating the 4 groups was very low in amyloid positive participants. On the other hand, NfL and GFAP separated Vas‐CU and VCI groups from CI reasonably well in the amyloid negative group.

**Conclusion:**

Plasma biomarkers associated with SVD‐burden provided reliable discriminative power in predicting VCID, particularly in the absence of amyloid, suggesting their utility in the prognosis and stratification for clinical trials.